# Quantification of Carotenoids, α-Tocopherol, and Ascorbic Acid in Amber, Mulligan, and Laird’s Large Cultivars of New Zealand Tamarillos (*Solanum betaceum* Cav.)

**DOI:** 10.3390/foods9060769

**Published:** 2020-06-11

**Authors:** Tung Thanh Diep, Chris Pook, Elaine C. Rush, Michelle Ji Yeon Yoo

**Affiliations:** 1 School of Science, Faculty of Health and Environment Sciences, Auckland University of Technology, Private Bag 92006, Auckland 1142, New Zealand; tung.diep@aut.ac.nz; 2 Riddet Institute, Centre of Research Excellence, Private Bag 11 222, Palmerston North 4442, New Zealand; elaine.rush@aut.ac.nz; 3 The Liggins Institute, The University of Auckland, Private Bag 92019, Auckland 1142, New Zealand; chris.pook@auckland.ac.nz; 4 School of Sport and Recreation, Faculty of Health and Environment Sciences, Auckland University of Technology, Private Bag 92006, Auckland 1142, New Zealand

**Keywords:** tamarillo, dietary antioxidants, β-carotene, ascorbic acid, α-tocopherol, carotenoids, provitamin A

## Abstract

Amber (yellow), Laird’s Large (red) and Mulligan (purple–red) cultivars of New Zealand tamarillo fruit were separated into pulp (endo- and mesocarp) and peel (exocarp), and analyzed by liquid chromatography-mass spectrometry/mass spectrometry (LC-MS/MS) for carotenoids, α-tocopherol and ascorbic acid contents. Fresh Mulligan pulp had the highest content of β-carotene (0.9 mg/100 g), α-tocopherol (1.9 mg/100 g), and ascorbic acid (28 mg/100 g). Higher concentrations of β-carotene and ascorbic acid, and lower concentrations of α-tocopherol were detected in pulps compared with peels. Compared with standard serves of other fruit, tamarillo had the highest β-carotene (9–20% RDI (recommended dietary intake)/serve), high ascorbic acid (67–75% RDI/serve), and α-tocopherol (16–23% adequate intake/serve). All cultivars had diverse carotenoid profiles dominated by provitamin A carotenoids (β-carotene and β-cryptoxanthin) and xanthophyll carotenoids (lutein; zeaxanthin and antheraxanthin). Favorable growth conditions (high light intensity and low temperature) may explain the higher antioxidant vitamin content in New Zealand tamarillos compared to those from other countries. Tamarillo peels may be used as natural food coloring agent to reduce waste and deliver sustainable production.

## 1. Introduction

Antioxidant compounds in fruit and vegetables reduce oxidative stress and these in turn contribute towards treatment and prevention of cardiovascular diseases and cancers, as demonstrated by many of biochemical and epidemiological studies [1]. Carotenoids (β-carotene, β-cryptoxanthin, lutein, and zeaxanthin), α-tocopherol and vitamin C are some of the most significant antioxidants present in fruit and vegetables [1]. Carotenoids are classified into two subgroups depending on their structure: carotenes (containing carbon and hydrogen atoms) and xanthophylls (containing at least one oxygen molecule) [2]. Carotenoids possess ability to trap singlet oxygen and eliminate peroxyl radical, thereby known as strong antioxidants. They act as photoprotectors to protect membrane lipids against reactive oxygen species due to the presence of double-conjugated links [3]. In vitro and in vivo studies have demonstrated the ability of carotenoids in prevention of cardiovascular diseases and protection against some types of cancers. Among carotenoids, β-carotene and β-cryptoxanthin own provitamin A activity and reduce the risk of cancer and coronary vein disease [3]. β-carotene has the highest vitamin A activity among provitamin A carotenoids, approximately twice that of α-carotene and β-cryptoxanthin [4]. β-carotene is an antioxidant which protects the body from several chronic illnesses caused by free radicals [2,5]. It also has demonstrated preventive and protective effects on apoptosis, fibrosis, hepatic steatosis, inflammation, and oxidative stress [2]. Meanwhile, β-cryptoxanthin is recognized as a strong antioxidant and is able to inhibit free radicals which can damage human cells and DNA [6].

Vitamin E is comprised of four tocotrienols and four tocopherols in α-, β-, δ- and γ- forms. Among these isomers, α-tocopherol has the highest biological activity because it is preferably absorbed and accumulated as the major vitamin E regulator in the humans. Other forms are degraded and excreted by the liver [7]. For several years, α-tocopherol has been recognized as a chief antioxidant that may have a role in the prevention of atherosclerosis due to its function as a scavenger of lipid peroxyl radicals, particularly, for oxidized low-density lipoprotein (LDL) [8]. Recent studies have examined other roles of α-tocopherol including pro-oxidant, cell signaling and gene regulatory functions together with antioxidant functions [8] but evidence for clinical effects is mixed. α-tocopherol has been used to treat several chronic, oxidative stress-induced pathologies and in the prevention and treatment of heart disease, cancer and Alzheimer’s disease [8].

Vitamin C or ascorbic acid has shown a wide range of biological functions including antioxidant activity, production of collagen, supporting wound healing, modulating immune responses and increasing non-haem iron absorption [9]. Through antioxidant function, vitamin C possibly reduces oxidative damage, which may lead to cancer, and has the ability to limit, in vivo; the formation of carcinogens, such as nitrosamines and modulate immune response [10]. This vitamin has been associated with reduced risk of cardiovascular disease since oxidative damage, including oxidative modification of low-density lipoproteins, is the major cause of cardiovascular disease [11,12]. It has been reported that vitamin C has synergetic interaction with other vitamins and plays a key role in regeneration of vitamin E [3].

Tamarillo (*Solanum betaceum* Cav.) belongs to the *Solanaceae* family genus *Solanum* along with tomatoes, capsicum and eggplant but it is distinctive for its carotenoid content [13]. Tamarillo is native to the Andean regions of South America [14]. Though it is grown in several countries (New Zealand, Colombia, Ecuador, Australia, USA, Africa and Asia), it is exclusively exported by New Zealand, Colombia and Australia [15]. Breeding and continuous re-selection of the tamarillo cultivars by New Zealand growers have led to three new and high-quality cultivars, colored yellow (Amber), red (Laird’s Large) and purple–red (Mulligan), which are currently grown commercially. The diversity of tamarillo color reflects the presence of either anthocyanins (purple and red), carotenoids (red, orange or yellow) and/or chlorophyll pigments. The pulp of the tamarillo, which constitutes 85% of the whole fresh fruit, is consumed fresh or preserved by pickling, stewing, or drying for later use as a condiment. Tamarillos are good sources of dietary fiber, vitamins A, C, and E, as well as some minerals, carotenoids and anthocyanins [14,16,17]. Several studies have attempted to explore proximate compositions [16,18], sugars and organic acids [14,19], pigments [20,21,22], phenolics and anthocyanins [23], carotenoids [20,24,25], vitamins [16,26] and minerals [27] in tamarillos. These studies have focused on the pulp of the tamarillo, with exclusion of peels.

To date, comparison of the phytochemical contents of Amber (yellow), Mulligan (purple–red) and Laird’s Large (red variety) cultivars remain unexplored. Furthermore, the antioxidant vitamins, carotenoid and pigment contents of tamarillos separated by peel (by-product) and pulp and what these values translate to, in terms of the recommended dietary intake (RDI) per serving, remain unknown. The aim of this study was to profile the pulp and peel of Amber, Larid’s Large, and Mulligan cultivars of tamarillos as potential dietary sources of provitamin A carotenoids, xanthophyll carotenoids, vitamin C, and vitamin E. Then, the RDI of these antioxidant vitamins from tamarillo was calculated and compared to other commonly consumed fruit. In addition, carotenoids and pigments in both peel and pulp of tamarillos were quantified to determine if the peel (by-product) of tamarillo could be used as a natural coloring agent.

## 2. Materials and Methods

### 2.1. Fruit Materials

Three kilograms each of Amber, Laird’s Large, and Mulligan tamarillo cultivars were obtained from the Northland region of New Zealand. This region has a latitude of 36° S and longitude of 174° E, temperatures of 22–26 °C in summer and 13–19 °C in winter and with regular irrigation provides the sub-tropical climate necessary for optimal growth of tamarillo [28]. Commercial maturity is between 21 and 24 weeks from anthesis. All three cultivars were grown under the same agricultural conditions following the guidelines of New Zealand Tamarillo Growers Association [28,29]. In summer, up to 10 L of water each day are required to ensure the ideal pH range of 5.8–6.5 in the fruit [29]. The macronutrients required for yields of 12–22 t/ha were 170 kg/ha of nitrogen, 45 kg/ha of phosphorus, 130–190 kg/ha of potassium, 65 kg/ha of calcium and 30 kg/ha of magnesium [29]. All the samples provided were authenticated as representative by New Zealand Tamarillo Growers Association.

All fruit were washed and dried. The physical dimensions and weights of each fruit were determined. The average raw mass of tamarillos was 90 ± 14, 108 ± 19 and 44 ± 4 g for Amber, Laird’s Large, and Mulligan cultivars, respectively. Twenty fruit were randomly chosen from a 3 kg batch of each cultivar (*n* = 20 × 3 cultivars). For the remaining whole fruit the average moisture content was determined by oven drying (Sanyo model MOV-112F, Sanyo Electric Co., Osaka, Japan) at 105 °C for 8 h and was for Amber, Laird’s Large, and Mulligan fruit 88, 88 and 89%, respectively. The 20 fruit from each cultivar were peeled manually using a peeler. The ratio of peel to pulp by weight was approximately 1:6. For each cultivar one peel and one pulp composite/aggregate sample was produced by thorough homogenization, snap-freezing with liquid nitrogen, lyophilization and grinding to powder then storage at −20 °C for further analysis. i.e., In all cases, three different samples of peel and pulp of the collected tamarillos were separately extracted and analyzed according to Food and Agricultural Organization guidelines for food composition data [30].

### 2.2. Chemicals, Reagents, and Standards

Optima LC-MS grade acetone, acetonitrile, acetic acid, ammonium (NH_4_), methanol, isopropanol and formic acid were from Thermo Fisher (Auckland, New Zealand). Tetrabromophenyl porphyrin (TBP), metaphosphoric acid, and analytical grade standards of α-tocopherol, β-carotene and ascorbic acid were obtained from Sigma-Aldrich (Auckland, New Zealand). Milli-Q water was produced by Purite Fusion Milli-Q water purifying machine (Purite Limited, Thame, Oxon, UK).

### 2.3. Analysis β-Carotene and α-Tocopherol in Tamarillos

The method of Gentili and Caretti [31] with some modifications was used for extraction. Dried, powdered samples (50 ± 0.5 mg) were extracted with 1 mL of acetone. The extract was vortexed for 30 s and incubated in the dark for 30 min at 4 °C with vortexing every 5 min, followed by centrifuging at 10,000 RCF for 10 min at 4 °C. A glass syringe was used to separate supernatant and the extract was transferred in a low volume vial insert inside an amber 1.8 mL glass vial, then stored at −20 °C until analysis.

**Figure 1:**
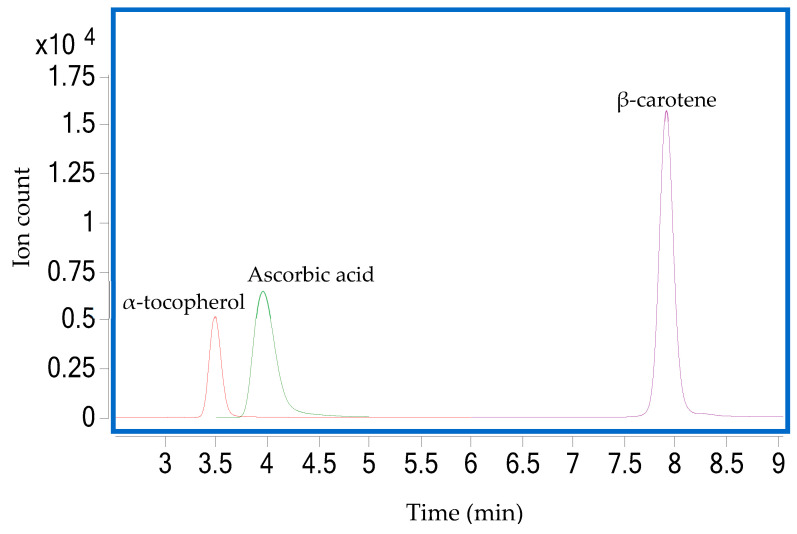
Multiple Reaction Monitoring (MRM) chromatograms of α-tocopherol, β-carotene and ascorbic acid standards.

Spectra of β-carotene and α-tocopherol were obtained by liquid chromatography-atmospheric pressure chemical ionization-mass spectrometry/mass spectrometry (LC-APCI-MS/MS) (Agilent Technologies Inc., Santa Clara, CA, USA) coupled with a diode array detector (DAD); and Agilent MassHunter Qualitative Analysis software (B.07.00) and Quantitative Analysis software for QQQ (B.07.01) (Santa Clara, CA, USA). The XSelect C18 column (100 × 2.1 mm, 3.5 μm; Waters, Ireland) was maintained at 50 °C. The mobile phases were 0.1% formic acid in Milli-Q as eluent A and pure methanol as eluent B. A gradient program was set up: 0–1 min, 5% of A; 1–9.2 min, 0% of A; 9.2–15 min, 5% of A. The flow rate was fixed at 0.4 mL/min and the injection volume was 5 μL. Double online detection was implemented in the DAD at 453 nm for β-carotene and 295 nm for both α-tocopherol and β-carotene. The MS was run in the negative mode. The MS collision energy was adjusted for each standard compound to produce product ion using a multiple reaction monitoring (MRM) mode which was applied to detect and quantify these analytes. The multimode (MMI) source was operated at APCI parameters as follows: gas temperature as 300 °C with a flow rate of 5 L/min. The capillary voltage and the nebulizer were set to 2.0–2.5 kV and at 50 psi, respectively. Vaporizer temperature was set at 250 °C. Quantification, quality control and validation of β-carotene and α-tocopherol were assured by the use of retention times, external standards and linearity of at least 6 point calibration curves of analyte standards (Supplementary Figure S1). Chromatograms of standard injections and a summary of the analytical method for each compound are presented in Figure 1 and Table 1, respectively. The sensitivity of the method was assessed by limit of detection (LOD) and limit of quantitation (LOQ) which were calculated based on the standard deviation of the response and the slope from the standard curve [32], and these are also presented in Table 1.

### 2.4. Analysis of Ascorbic Acid in Tamarillos

Extraction of ascorbic acid was performed according to the method described by Abushita, Hebshi, Daood and Biacs [33] with some modifications. Sample extracts were prepared, following the same procedure described in Section 2.3 but aqueous metaphosphoric acid (3%) was used instead of acetone. The liquid chromatography-electrospray ionization-mass spectrometry/mass spectrometry (LC-ESI-MS/MS) was used to determine vitamin C in peel and pulp of three tamarillo cultivars. The column was Poroshell 120 EC-C18 column (150 × 2.1 mm, 2.7 μm; Agilent, Santa Clara, CA, USA) and maintained at 22.6 °C. The mobile phase A was 0.1% formic acid in Milli-Q and mobile phase B was 0.1% formic acid in acetonitrile. The LC gradient was kept constant with 90% of A. The MS was run in the negative mode with the total time of 6.0 min. The injection volume and flow rate were 3.0 μL and 0.2 mL/min, respectively. The MMI source operating at ESI parameters were as follows: the gas temperature of 300 °C with a flow rate of 6 L/min. The capillary voltage and the nebulizer were set to 4.0 kV and at 15 psi, respectively. Two Agilent MassHunter software were used to qualify and quantify the ascorbic acid. Quantification of ascorbic acid in tamarillo extracts was implemented using standard calibration curves fitted with at least six suitable concentrations. Chromatogram of standard injections, a summary of analytical method and typical standard curve of ascorbic acid are presented in Figure 1, Table 1, and Figure S1, respectively.

### 2.5. Analysis of Other Carotenoid and Chrolophyll Pigments in Tamarillos

Preparation of sample extracts followed the same procedure described in Section 2.3, where acetone containing 1 mg/L TBP as internal standard was used as extraction solvent and the incubation time in the dark was 5 min. The LC-MS coupled with DAD, fluorescence detection (FLD) and a XSelect C18 column were used to separate and determine various pigments. The injection volume and total run time were 8.0 μL and 28.0 min, respectively. The solvents used were: (A) 0.1% formic acid in Milli-Q; (B) 0.1% acetic acid in acetonitrile containing 10 mM NH_4_; and (C) a mixture of 80% isopropanol, 20% acetonitrile and 0.1% acetic acid containing 10 mM NH_4_. The mobile phases were pumped at 0.3 mL/min with following gradient: 0–0.5 min, 50% of A and 50% of B; 0.5–10 min, 30% of A and 70% of B; 10–20 min, 3% of A and 97% of B; 20–20.20 min, 3% of A and 47% of B; 20.20–21 min, 3% of A and 97% of B; 21–28 min, 50% of A and 50% of B. Double detection was implemented in DAD between 300 and 640 nm. The MS was run in the positive ion mode. The MMI source operating at ESI were gas temperature of 300 °C, gas flow of 5.0 L/min, vaporizer temperature of 200 °C, nebulizer gas at 50 psi and capillary voltage of 2.0–2.5 kV. The pigments were determined by comparison with retention times from library and Agilent MassHunter Qualitative Analysis software was used to qualify these compounds. Background subtraction using the reference blank were implemented, then the peak areas of all compounds were normalized to that of the internal standard and the mass to estimate the relative abundance of pigments.

### 2.6. Statistical Analysis

All the analyses were conducted in triplicate on each sample and the results are presented as mean ± standard deviation (SD) which represents analytical variability (error) within sample. One-way analysis of variance (ANOVA) was applied to compare characteristics and intervarietal variability. Intervarietal differences were evaluated with Fisher’s (LSD) multiple comparison tests and are expressed as difference and 95% confidence interval of the difference. Data analysis was conducted using SPSS 25.0 (IBM Corp., Armonk, NY, USA) and the statistical significance level was set at *p* < 0.05.

## 3. Results

The LC-MS and the subsequent fragmentation of the predominant ion in MS-MS were used to identify β-carotene and α-tocopherol from the acetone extracts as well as vitamin C from aqueous metaphosphoric acid extract of peel and pulp of tamarillos. As shown in Figure 1, standard peaks of all antioxidant vitamins were ideal and showed accuracy and precision of the method. Further quantification of each identified analyte was carried out using a linear standard curve within a serial concentration range. Good correlations of all the analyzed compounds were achieved with R^2^ of the linearity >0.999 (Table 1). Also, the high sensitivity of the chromatography system and the method was confirmed through very low LOD (0.0258–0.8638 µg/L) and LOQ (0.0782–2.617 µg/L) (Table 1).

### 3.1. Concentration of β-Carotene in Tamarillos

**Figure 2:**
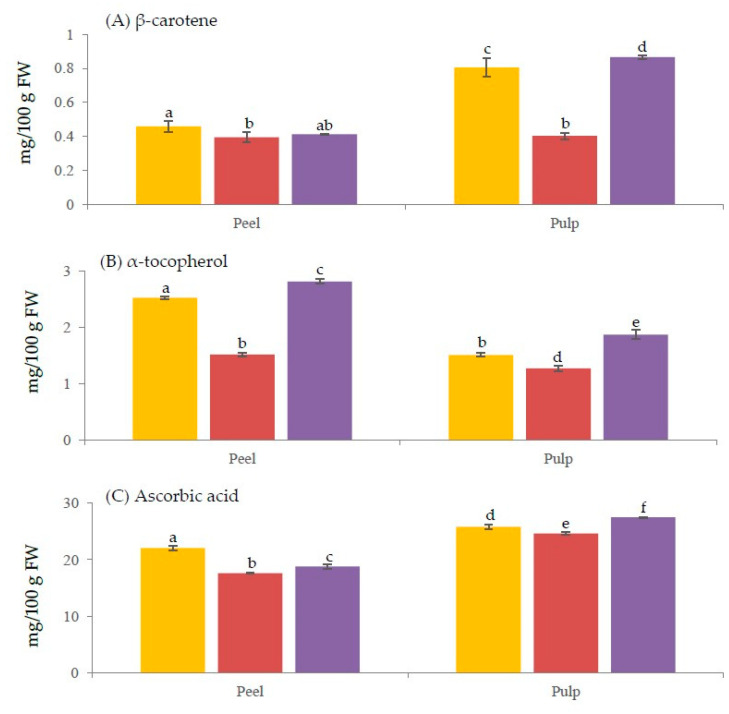
Average contents of β-carotene (**A**), α-tocopherol (**B**), and ascorbic acid (**C**) in the peel and pulp of three New Zealand tamarillo cultivars (■ Amber, ■ Laird’s Large, ■ Mulligan). Data are presented as mean (mg/100 g FW) and error bar (standard deviation) (*n* = 3). Different letters indicate statistical difference (*p* < 0.05).

Pulp of Laird’s Large fruit had less than a half the β-carotene of Amber and Mulligan cultivars (0.4 vs 0.8 and 0.9 mg/100 g of fresh weight (FW), respectively). Lairds Large pulp contained 47% and 50% of the β-carotene in Mulligan (difference 0.46 mg/100 g FW (95% CI 0.43, 0.49)) and in Amber (difference 0.4 mg/100 g FW (95% CI 0.32, 0.49)), respectively. The values obtained for the peels of all cultivars were similar, ranging from 0.4 to 0.5 mg/100 g FW (Figure 2A). The β-carotene content in pulp of Amber and Mulligan cultivars was almost a double of the peels, whereas both peel and pulp of Laird’s Large cultivar had similar amount of β-carotene. The pulp of Amber contained 177% more β-carotene than the peel (difference 0.35 mg/100 g FW 95% CI (0.25, 0.44)). Table 2 shows the contribution of tamarillos in relation to the RDI of the three antioxidant vitamins. The β-carotene per serve (120 g) [13] of the pulp of Amber, Laird’s Large, and Mulligan were 0.96, 0.48, and 1.08 mg, respectively which would supply 18, 9 and 20% of the RDI, respectively (Table 2). As Amber and Mulligan pulp possess greater than 10% of RDI for β-carotene, they could be considered to be the potential dietary sources.

### 3.2. Concentration of α-Tocopherol in Tamarillos

In contrast to the distribution of β-carotene, α-tocopherol was higher in the peel compared with the pulp of all three cultivars (1.2–1.7 times, *p* < 0.05) (Figure 2A,B). The peel and pulp of Mulligan showed the highest (2.8 and 1.9) and Laird’s Large peel and pulp the lowest (1.5 and 1.3 mg/100 g FW) content of α-tocopherol, respectively. Similar trends were observed for both β-carotene and α-tocopherol contents. Mulligan was the richest source of both compounds, followed by Amber, regardless of the tissue types. Lairds Large pulp contained 68% of the α-tocopherol in Mulligan (difference 0.6 mg/100 g FW (95% CI 0.45, 0.75)). The peel of Mulligan contained 150% more α-tocopherol than the pulp (difference 0.94 mg/100 g FW 95% CI (0.79, 1.09)). Laird’s Large, Amber and Mulligan cultivars could all be considered to be dietary sources (>10% Adequate Intake (AI)) of α-tocopherol, which met 16, 18 and 23% of AI, respectively (Table 2).

### 3.3. Concentration of Ascorbic Acid in Tamarillos

Significant differences have been detected in the ascorbic acid content in tissues and cultivars of tamarillo (Figure 2C). For the peels, the highest and the lowest contents of vitamin C were found in Amber (22 mg/100 g FW) and Laird’s Large (18 mg/100 g FW), respectively. The concentration of this vitamin in Mulligan peel was 19 mg/100 g FW. For the pulp, the Mulligan showed the highest vitamin C concentration (28 mg/100 g FW), followed by Amber (26 mg/100 g FW), and then Laird’s Large (25 mg/100 g FW). Mulligan pulp and Amber pulp contained 10% and 5% more ascorbic acid than Laird’s Large pulp (difference 2.85 mg/100 g FW 95% CI (2.45, 3.25) and difference 1.16 mg/100 g FW 95% CI (0.4, 1.94)), respectively. All pulp samples owned higher ascorbic acid than the peels, by approximately 15–30%. As shown on Table 2, one serve of Laird’s Large, Amber and Mulligan tamarillos supplied 30, 31.2 and 33.6 mg of ascorbic acid, respectively, meeting 67, 69 and 75% of the RDI, respectively.

### 3.4. Analysis of Other Carotenoids and Chlorophyll Pigments

Out of the 21 compounds analyzed, 20 pigments were detected in peel and pulp of the three tamarillo cultivars, except for chlorophyll D (Table 3). The major pigment in all pulp samples was β-carotene; found the lowest in Laird’s Large and more than twice as high in Mulligan. Zeaxanthin and β-cryptoxanthin were the highest in Amber peel and Mulligan peel, respectively (*p* < 0.05). β-carotene accounted for over 45% of the total pigment content in all pulp samples (Table S1). Meanwhile, the relative content of β-cryptoxanthin was similar between peels and pulps of all cultivars. Amber and Laird’s Large showed the highest (0.24–0.25) and the lowest (0.1–0.12 mg/100 g FW) relative contents of β-cryptoxanthin, respectively, regardless of the tissues (Table 3). This compound was the most dominant pigment in Mulligan peel (31.82%) as well as the second most abundant compound in the rest of the samples (over 13%) (Table S1).

Concentration of zeaxanthin was significantly different (*p* < 0.05) among all analyzed samples. It was noted that the relative concentration of zeaxanthin in peel was higher than in pulp (approximately 1.3–1.8 times), except for the Mulligan cultivar which had higher content of this pigment in pulp than in peel (Table 3). Zeaxanthin comprised 8.63–25.11 and 7.87–13.47% of total pigments in peels and pulps, respectively (Table S1). Concentration of lutein was higher in all pulps than in all peels; being the lowest and the highest in Laird’s Large peel (0.06 mg/100 g FW) and Mulligan pulp (0.2 mg/100 g FW), respectively. Antheraxanthin showed the highest and the lowest relative contents (0.15 and 0.07 mg/100 g FW) in Amber and Mulligan, respectively, for the peels. By contrast, the highest and the lowest constitutes of this pigment were found in Mulligan and Laird’s Large for the pulp (0.04 and 0.11 mg/100 g FW), respectively (Table 3). The percentage concentration (%) of lutein and antheraxanthin in all the analyzed samples were over 5% (Table S1).

Apart from these major pigments, tamarillos also contained other xanthophyll compounds including astaxanthin, violaxanthin, diadinoxanthin, dinoxanthin, flavoxanthin A, siphonaxanthin, and flavoxanthin B. The relative contents of these pigments varied between cultivars and tissues of tamarillo (*p* < 0.05), except for siphonaxanthin (*p* > 0.05). Higher relative content to total pigments of astaxanthin, violaxanthin, diadinoxanthin, and siphonaxanthin had been found in all peels than in all pulps. In contrast, all pulps showed higher relative content of dinoxanthin, compared to that of the peels. For flavoxanthin A, pulps of Laird’s Large and Mulligan showed higher content than in peels, whereas Mulligan pulp had lower content than the peel of the same cultivar for flavoxanthin B (Table 3).

Four isomers of chlorophyll (A, C1, C2 and C3) were detected in New Zealand tamarillo with variations observed across tissue types and cultivars. Chlorophyll C1 was abundant (1.15–4.5%), except for the Amber peel, which had chlorophyll C2 as the dominant chlorophyll with 4.49% (Table S1). Relative contents of other pigments varied depending on the cultivars and tissues. Among these compounds, violaxanthin accounted for relatively high content in all peels, with 0.04 mg/100 g FW (Table 3). Phaeophytin, a derivative of chlorophyll, had been detected only in pulp samples of all three cultivars with 0.02, 0.05 and 0.07 mg/100 g FW in Amber, Laird’s Large, and Mulligan, respectively. This pigment is also considered to be the main pigment in Laird’s Large pulp with the relative percentage content of 6.46%, followed by the Mulligan (4.21%) and then the Amber (1.01%) (Table S1).

## 4. Discussion

To our knowledge, this is the first study to quantify carotenoids, α-tocopherol, ascorbic acid and chlorophylls in peel (exocarp) and pulp (endocarp and mesocarp) of three New Zealand tamarillo cultivars. The most important finding is that both peel and pulp of tamarillo contained significant quantities of these antioxidant vitamins. We have shown that tamarillo is a dietary source of β-carotene and α-tocopherol (RDI and AI > 10%); and a good source of ascorbic acid (RDI > 25%). Among the pulp samples, Mulligan contained the most β-carotene, α-tocopherol and ascorbic acid. Atheraxanthin, β-carotene, zeaxanthin, β-cryptoxanthin, and lutein were the major (>5% of total) pigments in the three cultivars. From our findings, higher amounts of antioxidant vitamins would be supplied by consumption of the whole, unpeeled fruit rather than peeled.

Approximately 97% of total vitamin E is comprised of α-tocopherol in mature tomato [36], where a similar composition was expected in tamarillo of the same genus. Current findings of 1.9, 1.5 and 1.3 mg of α-tocopherol per 100 g FW in the Mulligan, Amber, and Laird’s Large pulps are in the same magnitude as the previous New Zealand-based research by Athar, McLaughlin and Taylor [37]. They have reported in 2003 of 1.9 mg/100 g FW in both yellow and red tamarillos. Lister, et al. [16] reported in 2005 that the gold tamarillo from New Zealand had higher content of tocopherols than the red one; 3.5 and 1.8 mg/100 g FW, respectively. New Zealand grown tamarillos are a good source of α-tocopherol (AI > 15%) compared to raw, peeled tomato (0.6 mg/100 g FW) and other common fruit [38]. α-tocopherol is one of the most common and biologically active compounds to reduce risks of some cancer and heart diseases, to supply dietary support for healthy lung, heart and digestive functions [39,40] as well as to protect the retina from light injury [41]. The Recommended Daily Allowance (RDA) of α-tocopherol is 15 mg for adult men and women, which is equivalent to α-tocopherol from about 6.5 serves of Mulligan tamarillo.

Ascorbic acid of 29.8–34.3 mg/100 g FW and 24.7 mg/100 g FW in pulp of red and gold varieties of New Zealand tamarillos have been reported [16,42]. Our results were lower than those values; however, they fell within the greater range for the red (19.3–41.6 mg/100 g FW) and the yellow cultivars (24.6–33.2 mg/100 g FW) [43]. The vitamin C concentrations in golden-yellow and purple–red tamarillos from Ecuador (17 and 16 mg/100 g FW, respectively) [14] and reddish-brown skin tamarillo from Malaysia (55.9 mg/100 g of dry weight (DW), approximately 8 mg/100 g FW) [26] were lower than the respective cultivars of New Zealand grown tamarillos. Compared to several other fruit, tamarillo met higher % of RDI of ascorbic acid than avocado, blueberry, boysenberry, feijoa, passionfruit and raspberry; and met similar % RDI to mandarin and lemon (Table 2) which are known as rich sources of vitamin C. Peels of three tamarillo cultivars showed higher ascorbic acid content than grapefruit, orange, and lemon peels with 113.3, 110.4, and 58.59 mg/100 g DW, respectively which are equal to 11, 15 and 6 mg/100 g FW, respectively [44].

The RDA for vitamin C is 75 and 90 mg for women and men, respectively [45] and this can be obtained from about 2.5 serves of Mulligan tamarillos. At moderate intake of 30–180 mg/day, vitamin C is absorbed approximately 70–90% [10]. Administration of 80 to 160 μM of ascorbic acid provided 34 and 61% protection against lipid peroxidation, respectively, and DNA damage was prevented by 95% with administration of 160 μM of ascorbic acid (*p* < 0.05) [46]. The co-administration of vitamin A (mainly β-carotene), E (mainly α-tocopherol) and vitamin C can reduce the incidence and delay the progression of several cancers, such as colon, esophagus, mammary gland, skin and stomach [47]. Also, during the lipid peroxidation and inhibition of inflammation, a synergetic interaction between ascorbic acid and α-tocopherol has been reported [7]. High contents of vitamin C, α-tocopherol (from current study) and rutin (from another study [23]) have been observed in tamarillo, therefore, further research should be conducted to investigate synergetic effect of vitamin C-rutin and vitamin C-α-tocopherol in tamarillo.

This is the first study to profile for carotenoids in peel and pulp of tamarillos from New Zealand. The presence of these main carotenoids (β-carotene, β-cryptoxanthin, zeaxanthin, lutein and antheraxanthin) had been reported by Rodriguez-Amaya, et al. [25] and then De Rosso and Mercadante [20] in Brazilian tree tomato; by Mertz, et al. [22] in Ecuadorian fruit; as well as by Giuffrida, et al. [21] in Colombian fruit. Violaxanthin had been found in tamarillo from Brazil [20]. However, this is the first time that astaxanthin; caricaxanthin; dinoxanthin; diatoxanthin; diadinoxanthin; flavoxanthin A; flavoxanthin B; fucoxanthin and siphonaxanthin were found in tamarillo. Consistent with findings by Mertz, et al. [22], the current research confirms that β-carotene was the most abundant carotenoid pigment in the unsaponified extracts of yellow and red tamarillo. By contrast, carotenoid profile in tamarillo from Brazil was dominated by β-cryptoxanthin (45.3%), followed by β-carotene (26.1%) zeaxanthin (5.1%), and antheraxanthin (4.0%) [20]. The researchers identified carotenoids in the whole tamarillo fruit, assuming that peel and pulp were eaten together. Also, the carotenoid compounds were determined in saponified samples. Mertz, et al. [22] reported β-carotene as the dominant carotenoid in unsaponified extracts, whereas β-cryptoxanthin as abundant carotenoid in saponified extracts. The difference may have come from the origin of fruit and method of extraction.

We have shown that pulp of the purple–red cultivar (Mulligan 0.9 mg/100 g FW) had more β-carotene than the yellow (Amber 0.8 mg/100 g FW), confirming the previous work (2009) in Ecuador sourced tamarillos, by Vasco, et al. [14]. In addition, the New Zealand Mulligan cultivar showed higher β-carotene concentration than reddish-brown skin tree tomato from Malaysia (4.80 mg/100 g DW, which is equivalent to 0.7 mg/100 g FW) [26]. The New Zealand grown Laird’s Large cultivar had slightly lower β-carotene content than red tamarillo from Ecuador with 0.51 mg/100 g FW [22]. The New Zealand Amber variety had almost twice the β-carotene than that reported for yellow tamarillos from Ecuador with 0.46 mg/100 g FW [22]. Similar to our findings, Athar, et al. [37] reported in 2003 that β-carotene contents were 0.600 and 0.763 mg/100 g FW in New Zealand red and yellow cultivars, respectively. Compared to other commonly consumed fruit, consumption of any of these tamarillo cultivars would contribute meaningfully to the dietary intake of β-carotene (Table 2). In addition, tamarillo peel showed higher content of β-carotene than mango peel and pomegranate peel; 3.16 and 2.47 mg/100 g DW, respectively, which are equivalent to 0.4 and 0.3 mg/100 g FW [48].

β-carotene acts as an inhibitor against lipoprotein oxidation [49] and compared to β-cryptoxanthin, doubled antioxidant activity had been found [2]. Further benefits over decreasing risks of cancers, cardiovascular disease and increasing immune response [5] are evident in the literature. For example, administration of β-carotene of 0.4, 0.8 and 1.6 μM reduced lipid peroxidation formation by 28, 31 and 46%, respectively; and 1.6 μM of β-carotene significantly (*p* < 0.05) decreased DNA damage by 91% [46]. One serve of Mulligan tamarillo contains 1.08 mg (approximately 2 μM) of β-carotene, which exceeds the dosage used in these studies.

We also report substantial quantities of the provitamin A (β-cryptoxanthin) in the New Zealand tamarillos. This hydrophilic carotenoid had been reported to be much better absorbed than other carotenoids [50]. The total concentration of β-cryptoxanthin (including all isomers) in saponified sample of whole tamarillo from Brazil was 2.1 mg/100 g FW [20]. In unsaponified extracts of red and yellow tamarillos from Ecuador, β-cryptoxanthin was present in 0.15 and 0.11 mg/100 g FW, respectively [22]. After saponification, the concentration of β-cryptoxanthin was 1.58 and 1.35 mg/100 g FW in red and yellow tamarillos, respectively [22]. This compound is more hydrophilic than α-carotene, β-carotene and lycopene, hence β-cryptoxanthin possesses higher absorbability than the others owing to its presence on the outer surface of micelles and higher solubility in the aqueous environment of the intestine [50]. During in vitro digestion, the percentage of β-cryptoxanthin incorporated into micelles was 3 times greater than that of β-carotene under the same conditions [51]. Compared to other common carotenoids, β-cryptoxanthin also owns greater bioaccessibility and bioavailability. A greater bioavailability of 725% has been reported in β-cryptoxanthin–rich foods than in β-carotene–rich foods [6]. Another study showed that β-cryptoxanthin from papaya had been 2.9 times more bioavailable than β-carotene from similar fruit [52].

Two isomers, lutein, and zeaxanthin, had also been recognized as antioxidants and they are known to inhibit cardiovascular disorders and some cancers [53]. However, the role of these pigments in eye health had received more attention from researchers since they accumulate in retina and lens eyes [49]. They act as a natural sunblock by absorbing excess light energy, which is believed to protect human eyes from harmful blue light. A considerable reduction of cataract to over 20% and macular degeneration to over 40% from high intake of lutein and zeaxanthin had been explored [54]. Poiroux-Gonord, Bidel, Fanciullino, Gautier, Lauri-Lopez and Urban [55] explored that a supra-additive protection of low-density protein occurs when lutein (a hydrophobic antioxidant) is associated with hydrophilic antioxidants such as rutin. Other xanthophylls found in tamarillo also had their functional roles reported in the literature. For example, astaxanthin has been recognized as an excellent antioxidant with an ability to suppress free radicals in their standard or stimulated form [55]. This compound had been reported as possessing significant antioxidant power with 10 and 500 times higher than β-carotene and vitamin E, respectively [56].

Similar to this study, chlorophyll pigments in tamarillos from Brazil, Ecuador and Colombia had been reported by De Rosso and Mercadante [20]; Mertz, et al. [22]; and Giuffrida, et al. [21], respectively. Chlorophyll A had been reported in tamarillos from different sources [27]; however, this is the first time that chlorophyll C1; chlorophyll C2; chlorophyll C3; and especially phaeophytin, were detected in tamarillo. Phaeophytin is the primary electron acceptor of Photosystem II in plants [57]. Acosta-Quezada, et al. [27] had found that chlorophyll *b* was the major chlorophyll in different tamarillo varieties. Chlorophyll may provide a darker flesh color, and this may be favorable for the purple-fleshed tamarillo [27]. Pigments play an important role in photosynthesis and formation of flavor compounds and plant hormones that are associated with food acceptability [53]. In plants, a combination of carotene–chlorophyll and xanthophyll–chlorophyll normally occurs, which are responsible for a variety of colors in fruit and vegetables [58]. As fruit matures, the content of chlorophyll decreases, and lead to an increase in colored carotenoid pigments. This was shown by the content of chlorophylls in most of the analyzed samples being relatively low or even absent (chlorophyll D). It is noted that pigments not only contribute to the exocarp (peel) and endo- and mesocarp (pulp) fruit colorations but also to health benefits.

Environmental factors affecting the accumulation of β-carotene, α-tocopherol, vitamin C, and other bioactive compounds in fruit have been reported. During the growth stage, low temperature is normally favorable for the accumulation of phenolics, carotenoids and vitamin C in some fruit [55]. For example, a positive effect of low temperature on carotenoids (i.e., β-carotene and total carotenoid increase by 30–100% and up to 30%, respectively), vitamin C [55] and α-tocopherol contents [7] in tomatoes was observed. This also explains the significant carotenoids, α-tocopherol and ascorbic acid contents in New Zealand tamarillos which are grown from April to October with an average temperature of 10–18 °C. Another positive factor for accumulation of α-tocopherol [7], vitamin C and phenolic compounds [55], is high light intensity or good exposure to sunlight. This has been observed in tomatoes where ascorbic acid content increased by up to 30% [55]. The high intensity light of New Zealand and the height of tamarillo trees (up to 5 m) may explain the higher amounts of phenolic compounds and especially vitamin C in tamarillo grown in New Zealand compared to other countries. This light exposure may enhance photooxidative stress or increase photosynthesis in tamarillo. Up to now, optimum growth conditions to optimize the biosynthesis and storage of β-carotene, vitamin C and α-tocopherol, in tamarillo remain unknown. Therefore, further research should be implemented to fill in the gap of the literature.

There were some limitations in comparing our study to previous reports. These include country of origin of the fruit, growing conditions (weather), methods of extraction, and analytical methods and the limited sample of fruit being analyzed. For example, aqueous acetone (80%) and spectrophotometry technique were used to extract and quantify chlorophyll concentration by Acosta-Quezada, et al. [27]. De Rosso and Mercadante [20] analyzed carotenoid pigments in the whole fruit rather than separating the tissues to peel and pulp. Lister, et al. [16] and Sivakumaran, et al. [42] calculated the vitamin E content based on the sum of α-, β-, γ- and δ-tocopherols. Also, both research groups used the standard methods published by the New Zealand Food Composition Database which have identified the vitamin C content from L-ascorbic acid plus L-dehydroascorbic acid.

Strengths include that to the best of our best knowledge, this is the first and the most comprehensive analysis of the antioxidant vitamins (A, E, C), carotenoids, and pigments composition of New Zealand tamarillo cultivars, with differentiation between peel and pulp tissues. Our results have demonstrated that tamarillo, both pulp and peel, is good a dietary source of carotenoid pigments (lutein and zeaxanthin), provitamin A (β-carotene and β-cryptoxanthin), vitamin C, and vitamin E (α-tocopherol) and therefore it may contribute to resolve micronutrient deficiency in New Zealanders [59] and deliver health and nutritional benefits arising from the antioxidant vitamins. The current project used LC-MS/MS which is a more advanced and highly sensitive analytical instrument compared to high-performance liquid chromatography-mass spectrometry/mass spectrometry (HPLC-MS/MS) used in the previous studies. Advantages of using LC-MS/MS included simpler extraction process and a better separation of analytes in food matrices by with a smaller column (small internal diameter) than that of the HPLC [60].

Future work in determining the total antioxidant activity and bioavailability of phytochemicals found in tamarillo for their synergistic functional roles in in vitro cell lines, animal models, and human clinical studies would be advantageous. The majority of tamarillo is eaten fresh and the fruit is underused by the food industry. Preservation of tamarillos and fortification of the extracts may help to increase nutritional value of processed foods. Tamarillo peels, which are discarded as by-products, may have potential usages in the food industry as an antioxidant and also as a natural food coloring agent to pursue the principles of “green chemistry”; reduce food waste and promote sustainable production.

## 5. Conclusions

The current study showed that tamarillo is a promising, whole-food plant source of antioxidant vitamins and carotenoids. Tamarillos could make a significant contribution to achieve the RDI or AI of β-carotene and α-tocopherol, compared to other commonly consumed fruit. Extraction and further preservation of these antioxidant vitamins from tamarillo into a tablet form may have a potential as a stand-alone natural dietary supplement, particularly the peels, which are often discarded as by-product. Extracts of tamarillo peel could also be used as novel food additives to enhance the functional qualities of other foods and to reduce food waste and to enhance sustainable food production. Further investigation in epidemiological and clinical studies would be desirable to investigate the synergistic effects of these bioactive compounds in tamarillo.

## Figures and Tables

**Table 1:** Method validation for identification and quantification of α-tocopherol, β-carotene and ascorbic acid in peel and pulp of three New Zealand tamarillos.

Parameters	α-Tocopherol	β-Carotene	Ascorbic Acid
RT (min)	3.524	7.931	3.965
Precursor ion (*m/z*)	431.0	537.0	175.0
Product ion (*m/z*)	165.0	537.0	115.0
Collision energy (V)	32	1	8
Regression equation	y = 4122.59x + 437.03	y = x − 1741.49	y = 1063.14x − 506.73
Linear fit correlation coefficient^2^	0.9991	0.9998	0.9991
Calibration range (mg/L)	0.1563–20	0.1563–20	0.625–20
Limit of detection (μg/L)	0.1771	0.0258	0.8638
Limit of quantification (μg/L)	0.5366	0.0782	2.617
Ion mode	Positive	Positive	Negative

**Table 2:** Quantity (mg) and recommended dietary intake (RDI) of vitamins that would be supplied by one serve of different tamarillo cultivars and other commonly consumed fruit in New Zealand [13]. Data was calculated based on the concentrations of the pulp samples.

Fruit	Serving Size (g)	β-Carotene (mg/serve)	RDI of β-carotene	α-Tocopherol (mg/serve)	AI of α-Tocopherol	Ascorbic Acid (mg/serve)	RDI of Ascorbic Acid
Tamarillos							
Amber	120	0.96	**18%**	1.8	**18%**	31.2	**69%**
Laird’s Large	120	0.48	9%	1.56	**16%**	30	**67%**
Mulligan	120	1.08	**20%**	2.28	**23%**	33.6	**75%**
Avocado	85	0.041	1%	1.61	**16%**	6.2	**14%**
Blackcurrant	118	0.189	4%	0.94	9%	189	**420%**
Blueberry	157	0.012	0%	1.41	**14%**	6	**13%**
Boysenberry	133	0.4	7%	1.46	**15%**	12.1	**27%**
Feijoa	84	0.026	0%	0.15	2%	25.5	**57%**
Grapefruit	118	0	0%	–	–	47.2	**105%**
Kiwifruit	100	0.049	1%	1.7	**17%**	122	**271%**
Lemon	65	–	–	–	–	33.8	**75%**
Mandarin	150	0.478	9%	0.68	7%	31.5	**70%**
Passion fruit	18	0.002	0%	–	–	3.6	8%
Persimmon	83	0.058	1%	0	0%	42.2	**94%**
Raspberry	136	0	0%	0.48	5%	18.5	**41%**
Strawberry	175	0.01	0%	0.72	7%	79.8	**177%**

RDI: recommended dietary intake [34]. AI: Adequate Intake (used when an RDI cannot be determined) 1 µg retinol (vitamin A) equivalent = 6 µg all trans β carotene RDI = 900 retinol equivalents. Vitamin E: AI = 10 mg, UL = 300 mg. Vitamin C: Estimated average requirement (EAR) = 30 mg, RDI = 45 mg. – : not identified. Bolded numbers are dietary source (>10% RDI) and bolded and underlined numbers are good source (>25% RDI) [35].

**Table 3:** Pigment compounds and their relative contents identified in the pulp and peel of three tamarillo cultivars. The results are presented as mean ± SD (*n* = 3) and listed in the order of bioactive groups. Alphabets indicate statistical difference (*p* < 0.05) across each row.

Pigments	Relative Concentration (mg/100 g FW)					
	Amber Peel	Amber Pulp	Laird’s Large Peel	Laird’s Large Pulp	Mulligan Peel	Mulligan Pulp
Provitamin A carotenoids						
β-Carotene	0.23 ± 0.07 ^a^	0.72 ± 0.19 ^b^	0.15 ± 0.02 ^a^	0.36 ± 0.1 ^a^	0.15 ± 0.03 ^a^	0.89 ± 0.2 ^b^
β-Cryptoxanthin	0.25 ± 0.08 ^a^	0.24 ± 0.04 ^a^	0.1 ± 0.01 ^b^	0.12 ± 0.03 ^b^	0.23 ± 0.05 ^a^	0.22 ± 0.04 ^a^
Xanthophyll carotenoids						
Astaxanthin	0.02 ± 0.01 ^a^	<0.005 ^b^	0.01 ± 0 ^b^	<0.005 ^b^	<0.005 ^b^	<0.005 ^b^
Violaxanthin	0.04 ± 0.02 ^a^	0.02 ± 0.01 ^a,b^	0.04 ± 0.02 ^a^	0.01 ± 0 ^b^	0.04 ± 0.01 ^a^	0.02 ± 0.01 ^a,b^
Diadinoxanthin	0.01 ± 0.01 ^a^	0.01 ± 0 ^a,b^	0.01 ± 0.01 ^a,b^	<0.005 ^b^	0.01 ± 0.01 ^a,b^	0.01 ± 0 ^a,b^
Antheraxanthin	0.15 ± 0.03 ^a^	0.09 ± 0.02 ^b,c^	0.09 ± 0.04 ^b^	0.04 ± 0.01 ^c^	0.07 ± 0.03 ^b,c^	0.11 ± 0.03 ^a,b^
Dinoxanthin	<0.005 ^a^	<0.005 ^a^	<0.005 ^a^	<0.005 ^a^	<0.005 ^a^	<0.005 ^b^
Lutein	0.13 ± 0.02 ^a,b,c^	0.17 ± 0.09 ^b,c^	0.06 ± 0.02 ^a^	0.08 ± 0.03 ^a^	0.09 ± 0.03 ^a,b^	0.2 ± 0.06 ^c^
Flavoxanthin A	0.02 ± 0 ^a,b^	0.02 ± 0.01 ^a,b^	0.01 ± 0 ^c^	0.02 ± 0.01 ^a^	0.01 ± 0.01 ^a,c^	0.03 ± 0 ^b^
Diatoxanthin	<0.005 ^a^	<0.005 ^a,b^	n.d	n.d	n.d	<0.005 ^b^
Zeaxanthin	0.32 ± 0.06 ^a^	0.24 ± 0.08 ^ab^	0.11 ± 0.07 ^c,d^	0.06 ± 0.02 ^d^	0.06 ± 0.01 ^d^	0.2 ± 0.06 ^b,c^
Siphonaxanthin	0.01 ± 0.01 ^a^	0.01 ± 0 ^a^	0.01 ± 0.01 ^a^	<0.005 ^a^	0.01 ± 0.01 ^a^	<0.005 ^a^
Caricaxanthin	n.d	<0.005 ^a^	<0.005 ^a^	n.d	n.d	n.d
Flavoxanthin B	0.01 ± 0 ^a^	0.01 ± 0.01 ^a,b^	<0.005 ^a^	0.01 ± 0.01 ^a,b^	0.01 ± 0 ^b^	<0.005 ^a^
Fucoxanthin	n.d	<0.005 ^a^	n.d	<0.005 ^a^	n.d	n.d
Chlorophyll pigments						
Chlorophyll A	n.d	<0.005 ^a^	<0.005 ^b^	<0.005 ^b^	<0.005 ^b^	<0.005 ^b^
Chlorophyll C1	0.01 ± 0.01 ^a^	0.02 ± 0.01 ^a^	0.03 ± 0.02 ^a^	0.01 ± 0.01 ^a^	0.02 ± 0.01 ^a^	0.02 ± 0.02 ^a^
Chlorophyll C2	0.06 ± 0.01 ^a^	<0.005 ^b^	<0.005 ^b^	<0.005 ^b^	<0.005 ^b^	<0.005 ^b^
Chlorophyll C3	0.02 ± 0.01 ^a^	<0.005 ^b^	<0.005 ^b^	<0.005 ^b^	n.d	<0.005 ^b^
Chlorophyll D	n.d	n.d	n.d	n.d	n.d	n.d
Phaeophytin	n.d	0.02 ± 0.01 ^a^	n.d	0.05 ± 0.01 ^a^	n.d	0.07 ± 0.05 ^a^

n.d: not detected. Means with different superscripts ^a,b,c,d^ are significantly different at *p* < 0.05.

## Supplementary Materials

The following are available online at https://www.mdpi.com/2304-8158/9/6/769/s1, Figure S1: Standard curves of α-tocopherol, β-carotene and ascorbic acid detected in tamarillo using Agilent Chemstation Software (Agilent Technologies, Australia) for LC-MS/MS; Table S1: Pigment compounds and their relative percentage contents identified in the pulp and peel of three tamarillo cultivars. The results are presented as mean ± SD and listed in the order of Retention Time (RT). Alphabets indicate statistical difference (*p* < 0.05) across each row.
