# Supplementary material for: Quantification of Carotenoids, α-Tocopherol, and Ascorbic Acid in Amber, Mulligan, and Laird’s Large Cultivars of New Zealand Tamarillos (Solanum betaceum Cav.)
Source: Foods. 2020 Jun 11;9(6):769. doi: 10.3390/foods9060769 (PMC7353566; doi:10.3390/foods9060769)
Supplement: Supplementary file 1 [file foods-09-00769-s001.pdf]

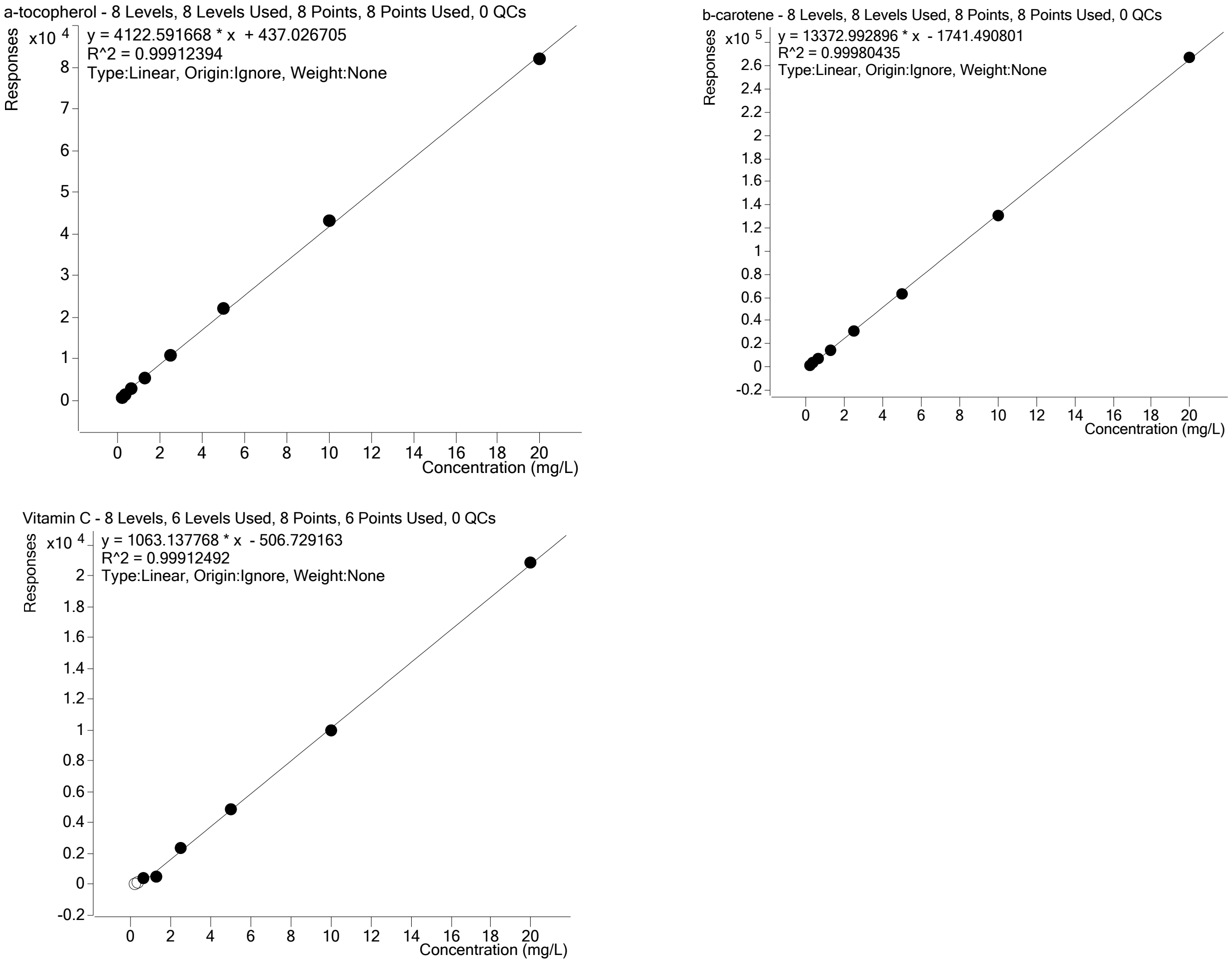

Figure S1. Standard curves of α-tocopherol, β-carotene and ascorbic acid detected in tamarillo using Agilent Chemstation Software (Agilent Technologies, Australia) for LC-MS/MS

Table S1. Pigment compounds and their relative percentage contents (%) identified in the pulp and peel of three tamarillo cultivars. The results are presented as mean ± SD and listed in the order of

bioactive groups, then retention time (RT). Alphabets indicate statistical difference ($P < 0.05$) across each row.

Pigments	RT (min)	*m/z*	Relative percentage contents (%)					
			Amber peel	Amber pulp	Laird's Large peel	Laird's Large pulp	Mulligan peel	Mulligan pulp
Provitamin A carotenoids								
β-Carotene	22.7	536.4	17.68 ± 4.08[a]	45.62 ± 10.45[b]	22.72 ± 4.02[a]	47.23 ± 7.71[b]	21.32 ± 4.38[a]	49.79 ± 9.64[b]
β-Cryptoxanthin	20.3	522.4	18.73 ± 2.85[a]	13.61 ± 2.76[a]	16.36 ± 4.29[a]	16.62 ± 5.29[a]	31.82 ± 3.3[b]	14.02 ± 1.88[a]
Xanthophyll carotenoids								
Astaxanthin	6.4	596.4	1.75 ± 0.89[a]	0.07 ± 0.06[b]	0.96 ± 0.58[ac]	0.03 ± 0.05[b]	0.49 ± 0.18[bc]	0.18 ± 0.16[bc]
Violaxanthin	9.7	600.4	3.01 ± 0.96[a]	1.18 ± 0.56[a]	6.79 ± 3.6[b]	1.2 ± 0.65[a]	6.25 ± 1.65[b]	1.24 ± 1.01[a]
Diadinoxanthin	11.4	583.4	1.06 ± 0.39[ab]	0.62 ± 0.08[ab]	1.66 ± 1.4[a]	0.36 ± 0.22[b]	1.44 ± 0.54[a]	0.65 ± 0.11[ab]
Antheraxanthin	11.4	584.4	11.8 ± 1.1[ab]	4.95 ± 1.12[c]	13.78 ± 5.22[a]	5.41 ± 1.04[cd]	10.31 ± 4.69[abd]	6.67 ± 1.21[bcd]
Dinoxanthin	12.5	642.4	0.02 ± 0.02[a]	0.04 ± 0.03[a]	0.03 ± 0.03[a]	0.04 ± 0.03[a]	0.03 ± 0.03[a]	0.15 ± 0.13[b]
Lutein	12.9	568.4	10.49 ± 0.74[a]	9.76 ± 5.07[a]	13.63 ± 6.64[a]	8.41 ± 2.45[a]	12.85 ± 4.55[a]	12.58 ± 3.96[a]
Flavoxanthin A	12.9	584.4	1.45 ± 0.64[ab]	1.07 ± 0.53[ab]	0.75 ± 0.57[a]	2.36 ± 1.22[b]	1.18 ± 0.79[ab]	1.82 ± 0.23[ab]
Diatoxanthin	13.0	566.4	0.27 ± 0.16[a]	0.08 ± 0.06[ab]	n.d	n.d	n.d	0.04 ± 0.04[b]
Zeaxanthin	13.0	568.4	25.11 ± 2.11[a]	13.47 ± 5.1[bc]	16.43 ± 8.73[b]	7.87 ± 2.02[c]	8.63 ± 1.18[bc]	12.62 ± 3.97[bc]
Siphonaxanthin	17.1	600.4	1.1 ± 1[ab]	0.45 ± 0.07[ab]	1.64 ± 1.27[a]	0.24 ± 0.2[b]	0.81 ± 0.79[ab]	0.11 ± 0.07[b]
Caricaxanthin	18.2	522.4	n.d	0.05 ± 0.04[a]	0.11 ± 0.1[a]	n.d	n.d	n.d
Flavoxanthin B	19.0	598.4	0.46 ± 0.06[ab]	0.55 ± 0.31[ab]	0.45 ± 0.28[ab]	1.32 ± 1.28[bc]	2.03 ± 0.36[c]	0.16 ± 0.13[a]
Fucoxanthin	21.1	658.4	n.d	0.03 ± 0.03[a]	n.d	0.05 ± 0.04[a]	n.d	n.d
Chlorophyll pigments								
Chlorophyll A	23.3	892.5	n.d	0.26 ± 0.16[a]	0.08 ± 0.06[b]	0.08 ± 0.07[b]	0.13 ± 0.11[ab]	0.05 ± 0.01[b]
Chlorophyll C1	18.5	610.2	0.84 ± 0.8[a]	1.15 ± 0.65[a]	4.5 ± 1.88[b]	2.1 ± 1.83[a]	2.62 ± 1[ab]	1.46 ± 1.18[a]
Chlorophyll C2	8.1	608.2	4.49 ± 0.12[a]	0.11 ± 0.06[b]	0.11 ± 0.08[b]	0.08 ± 0.07[b]	0.1 ± 0.06[b]	0.06 ± 0.05[b]
Chlorophyll C3	8.0	652.2	1.74 ± 0.87[a]	0.05 ± 0.04[b]	0.02 ± 0.01[b]	0.12 ± 0.09[b]	n.d	0.04 ± 0.02[b]
Chlorophyll D	21.6	894.5	n.d	n.d	n.d	n.d	n.d	n.d
Phaeophytin	23.8	896.8	n.d	1.01 ± 0.44[a]	n.d	6.46 ± 1.74[b]	n.d	4.21 ± 2.88[ab]

n.d: not detected
